# Regulation of TMEM16A by CK2 and Its Role in Cellular Proliferation

**DOI:** 10.3390/cells9051138

**Published:** 2020-05-05

**Authors:** Madalena C. Pinto, Rainer Schreiber, Joana Lerias, Jiraporn Ousingsawat, Aires Duarte, Margarida Amaral, Karl Kunzelmann

**Affiliations:** 1 Faculty of Sciences, University of Lisbon, BioISI—Biosystems & Integrative Sciences Institute, Campo Grande, 1749-016 Lisbon, Portugal; mdcpinto@fc.ul.pt (M.C.P.); jrlerias@fc.ul.pt (J.L.); amduarte@fc.ul.pt (A.D.); msamaral@fc.ul.pt (M.A.); 2 Physiological Institute, University of Regensburg, University Street 31, D-93053 Regensburg, Germany; rainer.schreiber@vkl.uni-regensburg.de (R.S.); jiraporn.ousingsawat@vkl.uni-regensburg.de (J.O.)

**Keywords:** TMEM16A, anoctamin 1, Ca^2+^ activated Cl^−^ channel, Casein kinase 2, CK2, cancer, proliferation

## Abstract

Casein kinase 2 (CK2) is a highly ubiquitous and conserved serine/threonine kinase that forms a tetramer consisting of a catalytic subunit (CK2α) and a regulatory subunit (CK2β). Despite being ubiquitous, CK2 is commonly found at higher expression levels in cancer cells, where it inhibits apoptosis, and supports cell migration and proliferation. The Ca^2+^-activated chloride channel TMEM16A shows similar effects in cancer cells: TMEM16A increases cell proliferation and migration and is highly expressed in squamous cell carcinoma of the head and neck (HNSCC) as well as other malignant tumors. A microscopy-based high-throughput screening was performed to identify proteins that regulate TMEM16A. Within this screen, CK2 was found to be required for proper membrane expression of TMEM16A. small interfering (si) RNA-knockdown of CK2 reduced plasma membrane expression of TMEM16A and inhibited TMEM16A whole cell currents in (cystic fibrosis bronchial epithelial) CFBE airway epithelial cells and in the head and neck cancer cell lines Cal33 and BHY. Inhibitors of CK2, such as TBB and the preclinical compound CX4549 (silmitasertib), also blocked membrane expression of TMEM16A and Ca^2+^-activated whole cell currents. siRNA-knockout of CK2 and its pharmacological inhibition, as well as knockdown or inhibition of TMEM16A by either niclosamide or Ani9, attenuated cell proliferation. Simultaneous inhibition of CK2 and TMEM16A strongly potentiated inhibition of cell proliferation. Although membrane expression of TMEM16A is reduced by inhibition of CK2, our data suggest that the antiproliferative effects by inhibition of CK2 are mostly independent of TMEM16A. Simultaneous inhibition of TMEM16A by niclosamide and inhibition of CK2 by silmitasertib was additive with respect to blocking cell proliferation, while cytotoxicity was reduced when compared to solely blockade of CK2. Therefore, parallel blockade TMEM16A by niclosamide may assist with anticancer therapy by silmitasertib.

## 1. Introduction

Casein kinase 2 (CK2) is a highly ubiquitous and conserved serine/threonine kinase that forms a tetramer consisting of a catalytic subunit (CK2α) and regulatory subunit (CK2β) [1]. CK2 phosphorylates hundreds of substrates. It contributes to a large number of cellular processes, but its main functions are related to cell growth, proliferation, and cell survival [2]. It supports cell proliferation and survival by antagonizing caspase activity and by potentiating survival signals. A multitude of mechanisms may contribute to these antiapoptotic functions [3]. A common inhibitor of CK2 that has been frequently used in previous studies is 4,5,6,7-tetrabromobenzotriazole (TBB) [4]. The orally bioavailable selective inhibitor of CK2, CX4945 (silmitasertib), has been shown to be antiproliferative and anti-angiogenic. It has the potential to be the first oral CK2 inhibitor that may advance from clinical trials to treatment of cancer patients [5,6].

TMEM16A belongs to a family of Ca^2+^-activated phospholipid scramblases and ion channels [7,8]. The 10 TMEM16 paralogs (ANO1-10; TMEM16A-K) are broadly expressed in epithelial and non-epithelial tissues [9]. TMEM16A is a Cl^−^ selective anion channel [10] with well-described functions in a number of tissues. TMEM16A is upregulated during cellular dedifferentiation and in cultured cells. It increases proliferation in many different tissues [11,12,13,14,15,16,17,18,19,20], and is expressed at high levels, particularly in head and neck cancers [15,16,21]. We recently identified niclosamide as a potent inhibitor of TMEM16A [22]. Niclosamide is an anthelminthic drug approved by the U.S. Food and Drug Administration that was also shown to inhibit Notch signaling [23], a pathway that is well known to participate in tumorigenesis [24]. A number of antineoplastic mechanisms of niclosamide have been described. Thus, niclosamide was shown to inhibit nuclear factor kappa B (NF-κB), Wnt/ß-catenin signaling, the IL-6-JAK1-STAT3-pathway, GSK-3 and more [25,26,27,28,29,30,31,32,33]. A recent paper suggests cell cycle arrest by niclosamide through activation of the Let-7d/CDC34 axis [34]. 

Niclosamide has been used in a number of preclinical studies and even in clinical trials with patients suffering from prostate and colorectal cancer [28,30,35,36,37,38,39]. Apart from various anti-cancer effects, niclosamide also inhibits the Ca^2+^-activated Cl^−^ channel TMEM16A. Blockade of TMEM16A is likely to take part in the inhibition of cell proliferation and cancer by niclosamide [15,16,40]. The present paper identifies a link between CK2 and TMEM16A, as CK2 supports membrane expression of TMEM16A. Both silmitasertib and niclosamide inhibited proliferation of head and neck cancer cells. Importantly, simultaneous application of both drugs strongly augmented their antiproliferative effects. The data suggest a combined treatment by silmitasertib and niclosamide to strongly augment anti-cancer potency of the individual drugs [40].

## 2. Material and Methods

### 2.1. Cell Culture

Cystic fibrosis bronchial epithelial cell lines (CFBE) were grown in minimum essential medium (MEM) supplemented with 2 mM glutamine. CFBE stably overexpressing 3HA-TMEM16A-eGFP were cultured in MEM supplemented with 2 mM glutamine, 2.5 μg/mL puromycin and 400 μg/mL G418. Cal33 and BHY cells, derived from head and neck carcinoma, were grown in DMEM without antibiotics, as described earlier [16]. All media were supplemented with 10% heat-inactivated fetal calf serum. All cells were cultured at 37 °C in a humidified atmosphere of 5% (*v/v*) CO_2_.

### 2.2. RT-PCR, siRNAs

Semi-quantitative RT-PCR was performed to detect the expression of TMEM16A and CK2 in CFBE and Cal33 cells. Total RNA was isolated using NucleoSpin RNA II columns (Macherey-Nagel, Düren, Germany). Total RNA (1 μg/50 μL reaction) was reverse-transcribed using random primers (Promega, Mannheim, Germany) and Reverse Transcriptase RNase H Minus (Promega, Mannheim, Germany). Each RT-PCR reaction contained sense and antisense primers for the respective gene (0.5 µM) or for GAPDH (0.5 µM), 0.5 μL cDNA and GoTaq Polymerase (Promega, Mannheim, Germany). After 2 min at 95 °C, cDNA was amplified during 30 cycles for 30 s at 95 °C, 30 s at 57 °C and 1 min at 72 °C. PCR products were visualized by loading on peqGREEN (Peqlab, VWR, Germany) containing agarose gels and analyzed using Meta Morph Version 6.2 (Molecular Devices, USA). The siRNAs—Silencer™ Select Negative Control siNEG1 (s813), siTMEM16A (HSS182856) and siCSNK2A2 (CK2α’, s3640) were purchased from ThermoFisher.

### 2.3. Patch Clamping

Cells grown on glass-coated cover slips were mounted on the stage of an inverted microscope (Zeiss, Munich, Germany) and kept at 37 °C. Patch pipettes were filled with a cytosolic-like solution containing (mM) KCl 30, K-gluconate 95, NaH_2_PO_4_ 1.2, Na_2_HPO_4_ 4.8, EGTA 1, Ca-gluconate 0.758, MgCl_2_ 1.03, D-glucose 5, ATP 3, pH 7.2. Patch-clamp experiments were performed in the fast whole-cell configuration. The bath was perfused continuously with Ringer solution (mM): NaCl 145, KH_2_PO_4_ 0.4, K_2_HPO_4_ 1.6, D-glucose 5, MgCl_2_ 1, Ca-gluconate 1.3, pH 7.4, containing 50 nM TRAM34 (Abcam, ab141885) at a rate of 8 mL/min. Patch pipettes had an input resistance of 2–4 MΩ and whole cell currents were corrected for serial resistance. Currents were recorded using a patch clamp amplifier (EPC 7, List Medical Electronics, Darmstadt, Germany), the LIH1600 interface and PULSE software (HEKA, Lambrecht, Germany) as well as Chart software (AD Instruments, Spechbach, Germany). In regular intervals, membrane voltage (Vc) was clamped in steps of 20 mV, from −100 to +100 mV from a holding voltage of −100 mV. Current density was calculated by dividing whole-cell currents by cell capacitance.

### 2.4. Western Blotting

Protein was isolated from CFBE and Cal33 cells using a sample buffer containing 50 mM Tris-HCl, 150 mM NaCl, 50 mM Tris, 100 mM dithiothreitol, 1% Nonidet P-40, 0.5% deoxycholate sodium and 1% protease inhibitor mixture (Sigma, Taufkirchen, Germany). Samples were separated by 7% SDS-PAGE and transferred to nitrocellulose membranes (GE Healthcare, Munich, Germany). Membranes were blocked with 5% NFM/TBST or 5% NFM/PBST at room temperature for 1 h and incubated overnight at 4 °C with rabbit monoclonal anti-DOG1 antibody (SP31, Novus, Braunschweig, Germany; 1:500, 1% NFM/TBST) and mouse monoclonal anti-Calnexin antibody (BD Biosciences; 1:5000, 5% NFM/PBST). The mouse anti-CK2α’ antibody (sc-514403, SantaCruz, USA) was used at 1:500 dilution. Subsequently, the membranes were incubated with HRP-conjugated goat anti-mouse or anti-rabbit, or donkey anti-goat IgG at RT for 2 h. Immunoreactive signals were visualized using a super-signal chemiluminescence substrate detection kit (Pierce Biotechonology, Rockford, IL, USA).

### 2.5. Immunocytochemistry

Cells were grown on glass coverslips and fixed with methanol and acetone (4:1) for 10 min at −20 °C. After washing 3 times with PBS supplemented with CaCl_2_ (0.7 mM) and MgCl_2_ (1.1 mM), cells were blocked with 3% bovine serum albumin (BSA) in PBS for 30 min at room temperature (RT), and incubated with anti-DOG1 primary antibody (1:200) in 1% BSA overnight at 4 °C. Binding of the primary antibody was visualized by incubation with a secondary antibody conjugated with Alexa 488 (1:500) in 1% BSA for 1 h at RT (Life Technologies, A-21206). Nuclei were stained with Hoechst 33342 (0.1 μg/mL PBS, Aplichem, Darmstadt, Germany). Cells were mounted on glass slides with mounting medium (DAKO Cytomation, Hamburg, Germany) and examined with an Axiovert 200 microscope equipped with ApoTome and AxioVision (Zeiss, Germany). Cellular distribution of endogenous TMEM16A was analyzed in CFBE or Cal33 cells in the presence or absence of CK2α’. Membrane and cytosolic expression were quantified in each cell by analyzing fluorescence intensities in the regions of interest (ROI) using the software ImageJ. Membrane regions were validated using high-resolution DIC image, that allowed us to clearly identify the plasma membrane of each cell.

### 2.6. Measurement of [Ca^2+^]_i_

Measurement of the global cytosolic Ca^2+^ changes were performed as described recently [41]. In brief, cells were loaded with 5 µM Fura-2, AM (Molecular Probes) in OptiMEM (Invitogen) with 0.02% pluronic (Molecular Probes) for 1 h at RT and 30 min at 37 °C. Fura-2 was excited at 340/380 nm, and the emission was recorded between 470 and 550 nm using a CCD-camera (CoolSnap HQ, Visitron Systems, Germany). Control of experiment, imaging acquisition and data analysis were done with the software package Meta-Fluor (Universal imaging, USA). [Ca^2+^]*_i_* was calculated from the 340/380 nm fluorescence ratio after background subtraction. The formula used to calculate [Ca^2+^]*_i_* was [Ca^2+^]*_i_* = *Kd* × (*R* − *R*_min_)/(*R*_max_ − *R*) × (S_f2_/S_b2_), where *R* is the observed fluorescence ratio. The values *R*_max_ and *R*_min_ (maximum and minimum ratios) and the constant S_f2_/S_b2_ (fluorescence of free and Ca^2+^-bound Fura-2 at 380 nm) were calculated using 1 µmol/liter ionomycin (Calbiochem), 5 µmol/liter nigericin, 10 µmol/liter monensin (Sigma) and 5 mmol/liter EGTA to equilibrate intracellular and extracellular Ca^2+^ in intact Fura-2-loaded cells. The dissociation constant for the Fura-2•Ca^2+^ complex was taken as 224 nmol/L.

### 2.7. Proliferation and Cell Death Assay

3-(4,5-dimethylthiazol-2-yl)-2,5-diphenyl-2H-tetrazolium bromide (MTT, M2128, Sigma-Aldrich, Taufkirchen, Germany), was dissolved in PBS to a final concentration of 5 mg/mL. The solution was filtered and stored at −20 °C, protected from the light. To determine proliferation, Cal33 cells or BHY cells (1.5 × 10^3^ cells) were seeded into 96-well plates and allowed to adhere overnight. The next day, cells were transfected with siRNAs (siTMEM16A, siCSNK2A2 or “scrambled” non-targeting siRNA) and/or treated with drugs (20 μM CX-4945, 0.5 μM niclosamide, 1 μM Ani9, 10 µM Eact or DMSO). Every 2 days, cells were again transfected and/or the medium with drugs was replaced, and experiments were performed. The medium was removed and 10 μL of MTT were added per well, together with 90 μL of new medium. MTT produces a yellowish solution that is converted to dark blue water-insoluble MTT formazan by mitochondrial dehydrogenases of living cells, therefore allowing the quantification of the living cells per well. After 2 h of incubation at 37 °C, the blue crystals were solubilized with DMSO and the intensity was measured colorimetrically at 570 nM using the plate reader NOVOstar (BMG Labtech, Offenburg, Germany).

### 2.8. Materials and Statistical Analysis

The CK2 inhibitors CX-4945 (silmitasertib) and TBB (4,5,6,7-Tetrabromobenzotriazole) were purchased from Cayman Chemicals and Sigma, respectively. Niclosamide was from Sigma (Germany). Data are reported as means ± SEM. Student’s *t*-test (for paired or unpaired samples as appropriate) or ANOVA were used for statistical analysis. A value of *p* < 0.05 was accepted as a significant difference.

## 3. Results

### 3.1. High-Throughput Assay Identifies CK2 as a Regulator of TMEM16A

**Figure 1:**
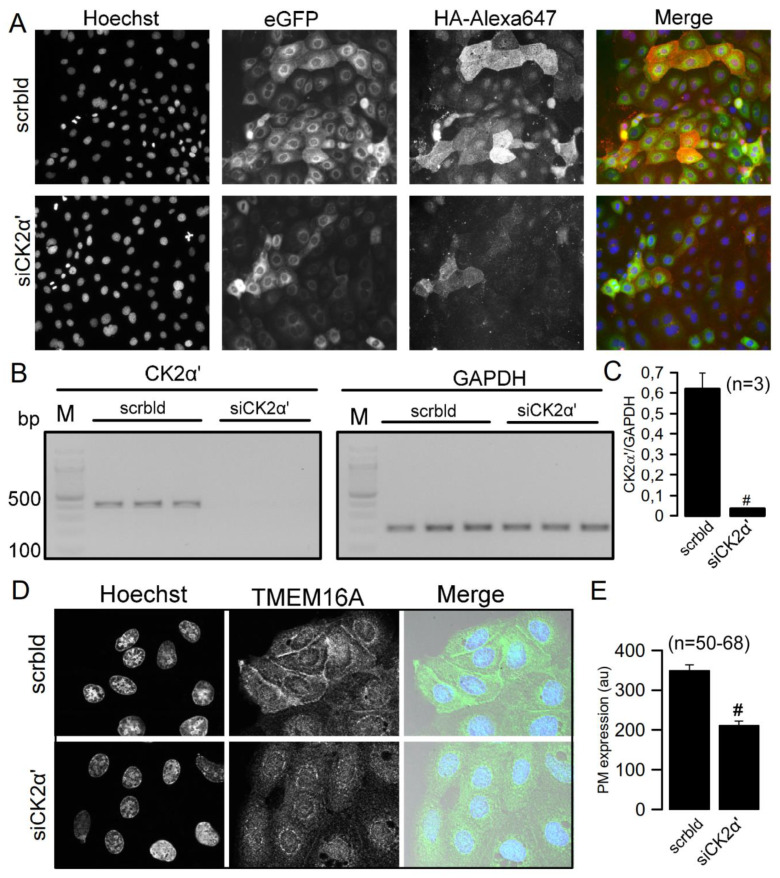
CK2 controls membrane expression of TMEM16A in CFBE airway epithelial cells. (**A**) Expression of double-tagged (eGFP and extracellular HA-tag) TMEM16A in CFBE airway epithelial cells. Membrane localized TMEM16A (Alexa647 positivity) was detected by an extracellular anti-HA-Alexa647-conjugated antibody. (**B**,**C**) RT-PCR and densitometric analysis indicating successful knockdown of CK2α’, #significant inhibition (unpaired *t*-test; *p* = 0.01). (**D**,**E**) Immunocytochemistry of TMEM16A expressed endogenously in CFBE cells. Membrane expression was reduced by knockdown of CK2α’, #significant inhibition (unpaired *t*-test; *p* = 0.000000002). Mean ± SEM. In parentheses are numbers of experiments.

A microscopy-based assay has been performed to identify novel regulators of the Ca^2+^-activated Cl^−^ channel TMEM16A [42]. siRNA screening for interactors of TMEM16A was performed in CFBE airway epithelia cells overexpressing double-tagged TMEM16A. CFBE cells were chosen because we intended to identify proteins that could be targeted in order to improve TMEM16A function, and thus Ca^2+^-dependent Cl^−^ secretion in cystic fibrosis airway epithelial cells [43]. We identified CK2 as a positive regulator of TMEM16A. Because TMEM16A is particularly known to be upregulated in head and neck squamous cell carcinomas (HNSCC), where CK2 also has a pro-cancerous role [43], we examined the hypothesis that CK2 promotes proliferation of the HNSCC cell lines Cal33 and BHY through activation of TMEM16A, which would have consequences for the treatment of HNSCC. siRNA-knockdown of the broadly expressed casein kinase 2 subunit CK2α’ was found to downregulate membrane expression of overexpressed TMEM16A containing a C-terminal green fluorescence protein (GFP) and an extracellular (human influenza hemagglutinin) HA tag (Figure 1A–C). Membrane expression was detected using an extracellular HA tag and binding of a fluorescent antibody to the extracellular HA tag. We examined whether endogenously expressed TMEM16A is equally regulated by CK2 and used CFBE cells that express only endogenous TMEM16A. Indeed, plasma membrane expression of endogenous TMEM16A was significantly inhibited upon knockdown of CK2α’ (Figure 1D,E). This effect of knockdown of CK2α’ was specific in as much as membrane expression of the common housekeeper ATPase Na^+^/K^+^-ATPase was not affected by the knockdown (Supplementary Figure S1).

### 3.2. Knockdown or Inhibition of CK2 Inhibits Activation of TMEM16A

**Figure 2:**
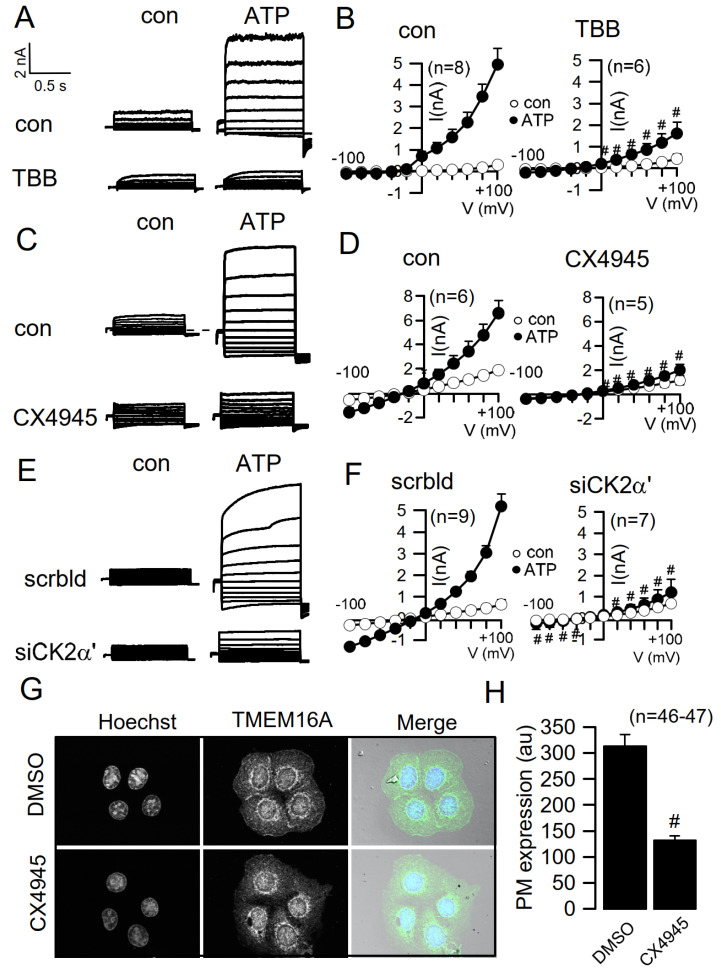
Inhibitors of CK2 inhibit TMEM16A in CFBE airway epithelial cells. (**A**–**F**) Whole cell current overlay recorded in patch clamp experiments and current/voltage relationships. ATP (100 µM) activated TMEM16A whole cell Cl^−^ currents that were strongly inhibited by the CK2-inhibitors TBB (10 µM; #significant inhibition, unpaired *t*-test; *p* = 0.01, (**A**,**B**)) and CX4945 (20 µM; #significant inhibition, unpaired *t*-test; *p* = 0.02; (**C**,**D**)), and siRNA-knockdown of CK2α’ (#significant inhibition, unpaired *t*-test; *p* = 0.0001; (**E**,**F**)). (**G**,**H**) Plasma membrane (PM) expression of endogenous TMEM16A in CFBE cells and inhibition of PM expression by the CK2-inhibitor CX4945 (#significant inhibition, unpaired *t*-test; *p* = 0.000000000007). Mean ± SEM #significant inhibition (*p* < 0.05; unpaired *t*-test). In parentheses are numbers of experiments.

TMEM16A is a Ca^2+^-activated Cl^−^ channel that is activated through stimulation of G-protein coupled receptors (GPRCs) that couple to phospholipase C, such as ATP-activated purinergic receptors. Stimulation of CFBE cells with extracellular ATP does increase intracellular Ca^2+^, which in turn will activate TMEM16A [42,44]. As shown in Figure 2, ATP activated TMEM16A whole cell currents in CFBE cells. Activation was strongly suppressed by preincubation of the cells for 30 min with the CK2 inhibitor TBB (Figure 2A). The summary of these experiments is shown in Figure 2B as current/voltage relationships of ion currents activated in control cells (left) and in TBB-treated cells (right). We also found that the CK2 inhibitor CX4945 suppressed ATP-induced whole cell currents even more potently than TBB (Figure 2C,D). In contrast, acute application of CX4945 to pre-activated TMEM16A did not clearly inhibit whole cell currents. Finally, knockdown of CK2α’ (siCK2α’) strongly attenuated TMEM16A currents stimulated by ATP (Figure 2E,F). Similar to knockdown of CK2α’ (Figure 1D), CX4945 also inhibited membrane expression of TMEM16A (Figure 2F,G).

### 3.3. CK2 Regulates Membrane Expression of TMEM16A in Cal33 Head and Neck Cancer Cells

**Figure 3:**
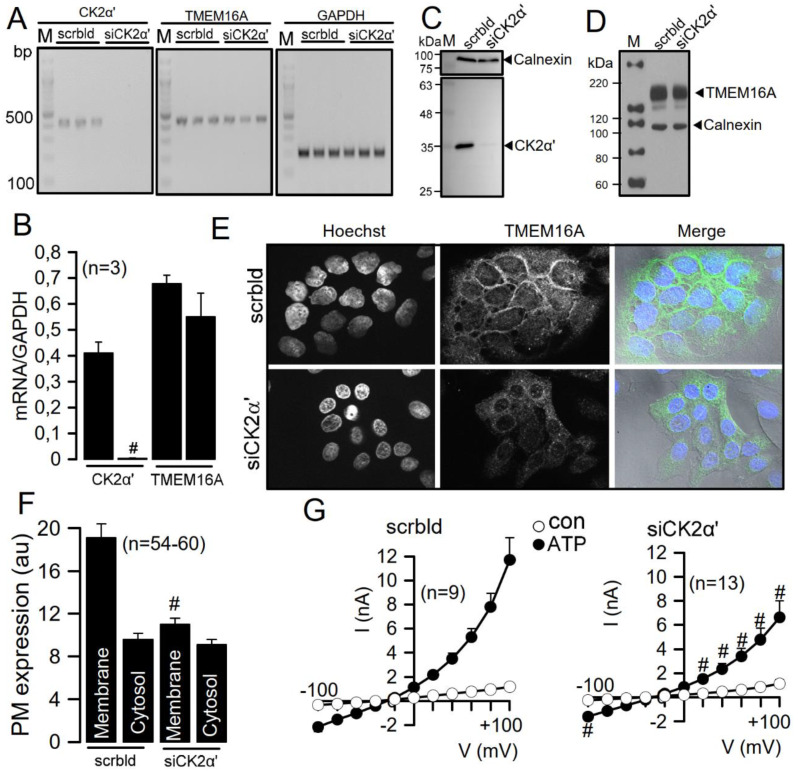
Role of CK2 for plasma membrane expression of TMEM16A in Cal33 head and neck cancer cells. (**A**,**B**) RT-PCR and densitometric analysis indicating successful knockdown of CK2α’ by siRNA for CK2α’ in Cal33 head and neck cancer cells (#significant inhibition, unpaired *t*-test; *p* = 0.01). Knockdown of CK2α’ did not inhibit transcription of TMEM16A. (**C**,**D**) Western blot analysis indicating successful knockdown of CK2α’ but unaffected expression of TMEM16A. (**E**,**F**) Plasma membrane (PM) expression of TMEM16A expressed endogenously in Cal33 cells and inhibition of PM expression by knockdown of CK2α’ (#significant inhibition, unpaired *t*-test; *p* = 0.00000002). (**G**) Current/voltage relationships of ATP-activated TMEM16A whole cells currents, indicating inhibition of TMEM16A by knockdown of CK2α’ (#significant inhibition, unpaired *t*-test; *p* = 0.01). Mean ± SEM. In parentheses are numbers of experiments.

TMEM16A is strongly expressed in head and neck cancer cells. The coding sequence of TMEM16A is located in the tumor-associated amplicon 11q13. High expression levels for TMEM16A correlate with poor survival of patients with head and neck cancers [16]. Our previous studies demonstrated the proliferative effect of TMEM16A in different head and neck cancer cell lines such as Cal27, Cal33 and BHY, as well as growth of soft tissue cancer in nude mice [15,16,40]. We therefore analyzed CK2-dependent regulation of TMEM16A-expression in Cal33 cells using siRNA for CK2α’, which potently suppressed CK2α’ mRNA as well as protein (Figure 3A–C). However, siRNA-knockdown of CK2α’ did not affect total expression of TMEM16A, as shown by Western blotting (Figure 3D). In contrast and similar to CFBE cells, knockdown of CK2α’ clearly reduced plasma membrane expression of TMEM16A in Cal33 cells (Figure 3E,F). Accordingly, TMEM16A currents activated by ATP were also inhibited by knockdown of CK2α’ (Figure 3G). However, attenuation of TMEM16A currents was less pronounced than in CFBE cells, which is due to excessive levels of TMEM16A-expression in Cal33 cells [16].

### 3.4. Inhibition of CK2 and TMEM16A Inhibits Cell Proliferation

**Figure 4:**
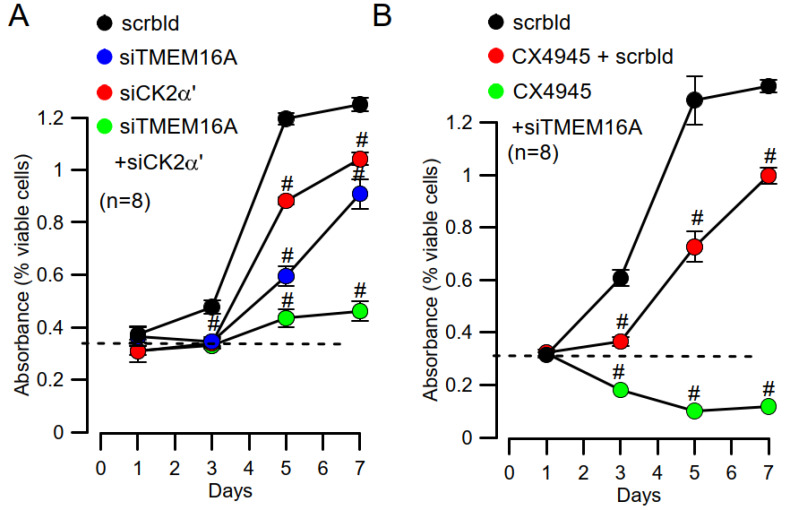
Inhibition of proliferation by knockdown of CK2α’ and TMEM16A. (**A**) Cell proliferation assessed in MTT assays and shown as absorbance. Both siRNA-knockdown of CK2α’ and TMEM16A inhibited cell proliferation (#significant inhibition, unpaired *t*-tests; *p* = 0.0001). Simultaneous knockdown of CK2α’ and TMEM16A had a more pronounced inhibitory effect on cell proliferation (#significant inhibition, unpaired *t*-test; *p* = 0.0015). (**B**) Inhibition of cell proliferation by the CK2-inhibitor CX4945 (20 µM) and additional inhibitory effect of TMEM16A-knockdown (#significant inhibition, unpaired *t*-test; *p* = 0.0001). Mean ± SEM. In parentheses are numbers of experiments.

Knockdown of TMEM16A attenuates cell proliferation [16], and this was also observed in the present study with Cal33 cells (Figure 4A). siRNA-knockdown of CK2α’ inhibited cell proliferation equally well. Notably, combined knockdown of both TMEM16A and CK2α’ had a more pronounced inhibitory effect on cell proliferation (Figure 4A). It suggests that CK2 and TMEM16A control cell proliferation in part by independent mechanisms. This was also found when CK2 was inhibited by CX4945 instead of siRNA-knockdown. CX5945 alone inhibited proliferation similar to siRNA-CK2α’, but CX4945 + siRNA-TMEM16A abolished proliferation completely (Figure 4B).

**Figure 5:**
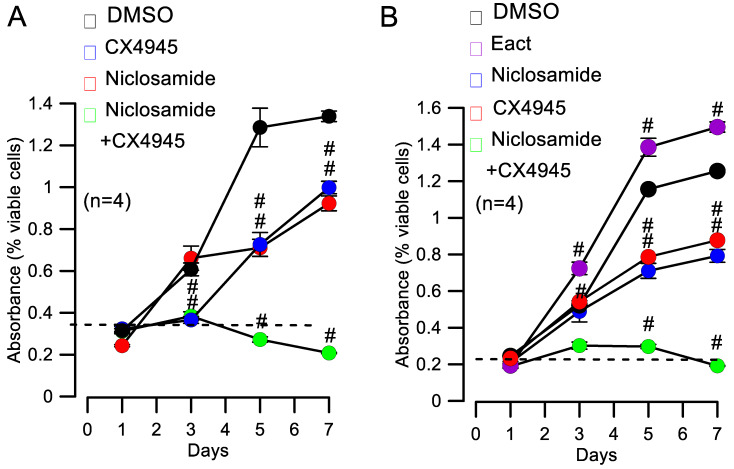
Blockers of CK2 and TMEM16A inhibit proliferation of Cal33 and BHY head and neck cancer cells. (**A**) Blocking CK2 by CX4945 (20 µM) and blocking TMEM16A by niclosamide (0.5 µM) inhibited proliferation of Cal33 cells. Simultaneous application of both blockers had an additive effect (#significant inhibition, unpaired *t*-tests; *p* = 0.0001). (**B**) Enhanced cell proliferation of BHY cells induced by the TMEM16A-activator, Eact. Blocking CK2 by CX4945 (20 µM) and blocking TMEM16A by niclosamide (0.5 µM) inhibited proliferation of BHY cells. Simultaneous application of both blockers had an additive effect (#significant inhibition, unpaired *t*-tests; *p* = 0.000015). Mean ± SEM. In parentheses are numbers of experiments.

As outlined above, niclosamide is a potent inhibitor of TMEM16A and an anticancer drug. It also inhibited proliferation of Cal33 cells in the present study (Figure 5A). Again, the combination of niclosamide together with CX4945 completely inhibited cell proliferation (Figure 5A). We performed similar studies in BHY cells, another head and neck cancer cell line [16], in order to validate the results obtained in Cal33 cells. Application of only CX4945 or niclosamide inhibited cell proliferation by about 50%. In contrast, simultaneous application of CX4945 and niclosamide essentially abolished proliferation (Figure 5B). Interestingly, the activator of TMEM16A, Eact [45], further augmented proliferation of BHY cells (Figure 5B).

### 3.5. Inhibition of TMEM16A and Inhibition of CK2 Attenuates Receptor-Mediated Increase in the Intracellular Ca^2+^ Concentration

**Figure 6:**
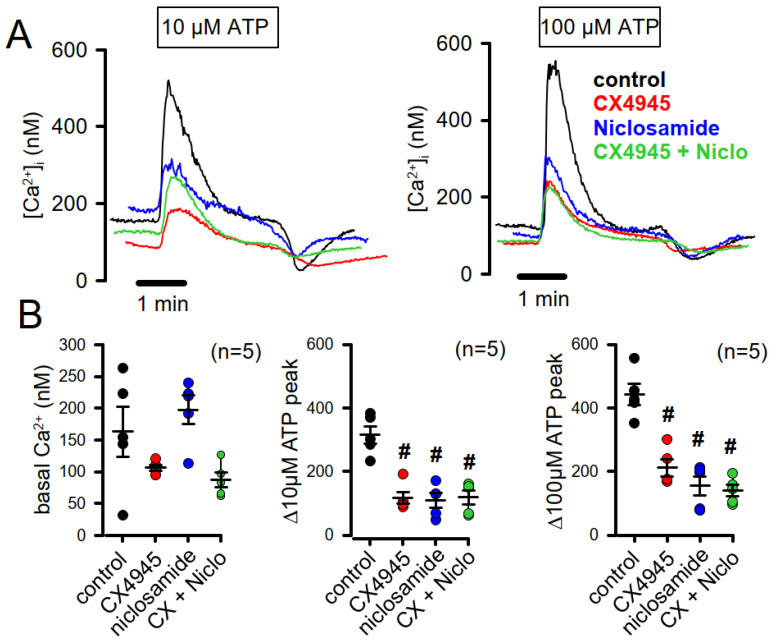
Blockers of CK2 and TMEM16A inhibit receptor-mediated Ca^2+^ signaling. (**A**,**B**) Original recordings and summaries for basal and ATP-induced intracellular Ca^2+^ concentrations in Cal33 cells. Increase of intracellular Ca^2+^ by 10 and 100 µM ATP, respectively. Both CX4945 (20 µM; #significant inhibition, ANOVA; *p* = 0.0004) and niclosamide (1 µM; #significant inhibition, ANOVA; *p* = 0.0002) largely reduced ATP-induced Ca^2+^ increase. Mean ± SEM. In parentheses are numbers of experiments.

TMEM16A has a pronounced impact on intracellular Ca^2+^ ([Ca^2+^]*_i_*) signaling, as reported earlier, which is explained by its interaction with the endoplasmic reticulum (ER) inositolphosphate receptor IP_3_R and possibly by the impact of TMEM16A-mediated Cl^−^ transport on Ca^2+^ signaling [40,46,47]. Because intracellular Ca^2+^ is a major regulator of cell proliferation, we examined if inhibition of TMEM16A by niclosamide exerts similar effects on intracellular Ca^2+^ signaling in Cal33 cells. Niclosamide did not change basal intracellular Ca^2+^ concentrations but strongly attenuated Ca^2+^ rise, induced by 10 and 100 µM ATP, respectively (Figure 6). It is notable that the CK2-inhibitor CX4945 also strongly reduced intracellular Ca^2+^ levels. This previously unrecognized effect of CX4945 on intracellular Ca^2+^ is likely to contribute to its antiproliferative/anticancer effects. Simultaneous inhibition of TMEM16A and CK2 did not further increase the inhibitory effect on [Ca^2+^]*_i_*. Taken together, blocking CK2 and TMEM16A inhibits cell proliferation, partially by overlapping mechanisms. Because inhibition of both pathways significantly augments inhibition of cell proliferation, it may be considered to use CX4945 and niclosamide simultaneously in patients with cancer.

## 4. Discussion

### CK2 and TMEM16A Regulate Cell Proliferation

In the present study, we have shown that the ubiquitous and constitutively active kinase CK2 controls membrane expression of the Ca^2+^-activated Cl^−^ channel, TMEM16A, in vitro. High-throughput screening was performed by stably expressing a TMEM16A construct (3HA-TMEM16A-eGFP) in CFBE cells, that contains a hemagglutinin tag (YPYDVPDYA) inserted in triplicate (3HA) between His^396^ and Asn^397^, i.e., in the first extracellular loop of TMEM16A. This extracellular HA-tag, if present, can be immuno-detected in non-permeabilized cells, as the antibody binds only to the plasma membrane-localized TMEM16A. Images were acquired using an automated widefield epifluorescence microscope. It means that cells were illuminated from above and the whole specimen was exposed to the light source, explaining this type of membrane staining [42]. The results identify TMEM16A as another ion channel that is regulated by CK2.

Earlier studies demonstrated that the cystic fibrosis transmembrane conductance regulator (CFTR) requires CK2 to be fully active [48,49,50,51]. We and others also demonstrated that CK2 positively regulates the epithelial Na^+^ channel, ENaC, which is important to control renal Na^+^ excretion [52,53]. For both CFTR and ENaC, consensus sides for CK2-dependent phosphorylation have been found. We searched for putative CK2 phosphorylation sites in human TMEM16A (abcd isoform) using PROSCAN/PROSITE databases, and identified 10 putative CK2 phosphorylation sites. Two stronger consensus CK2 sides were located at the N-terminus and one was located at the C-terminus of TMEM16A. The N-terminus is relevant for membrane trafficking. Its elimination abolished expression. Truncation of the C-terminus reduced ATP-activated whole cell currents in our previous report [54,55]. This could suggest a role of CK2 phosphorylation for activation of TMEM16A. However, it is currently unclear whether these sides are truly phosphorylated by CK2. For example, a serine is located at position 42 within the N-terminus and may possibly affect membrane targeting when phosphorylated. S42 phosphorylation could also change the interaction of TMEM16A with accessory proteins, such as the ezrin–radixin–moesin network [56].

A role of CK2 has been found for several other ion channels and transporters [51,57,58]. As for TMEM16A, also for CFTR and ENaC, CK2 was shown to support their intracellular processing and trafficking to the plasma membrane [52,59]. In this context, it is noteworthy that CK2 phosphorylates Sec31 and regulates ER-To-Golgi trafficking [60]. Also, peripheral steps of membrane fusion, exocytosis and insertion of proteins into the plasma membrane via the synaptosomal-associated protein receptor (SNARE) machinery is controlled by CK2 [61]. Transcription of TMEM16A is under the control of the transcription factor signal transducer and activator of transcription 6 (STAT6), while CK2 is known to affect STAT6 activity [62,63]. However, we did not find evidence for reduced expression of TMEM16A by inhibition of CK2. Taken together, inhibition of CK2 is likely to inhibit TMEM16A activity, in part by inhibition of plasma membrane expression and probably by inhibition of intracellular Ca^2+^ signaling [54].

An essential result of the present study is that co-application of niclosamide enhanced the anti-proliferative effect of CX4945 remarkably (Figure 5), but at the same time, lowered the cytotoxic (cell death) effect exerted by CX4945 (Supplementary Figure S2). The inhibitory effect of niclosamide on cell proliferation was validated by Ani9, another more specific inhibitor of TMEM16A (Supplementary Figure S3). Therefore, combined inhibition of CK2 and TMEM16A by CX4945 and niclosamide respectively, would maybe reduce the concentration of CX4945 required in a cancer patient. While our present data only demonstrate inhibitory effects of CX4945 in vitro, our previous experiments also demonstrated the role of TMEM16A for cancer growth in vivo [15,16]. Although CX4945 was used at µM concentrations in the present in vitro study, additional experiments show that it also inhibits proliferation at nanomolar concentrations (Supplementary Figure S4). Taken together, we may speculate that co-application of niclosamide together with CX4945 could allow for further reduction of the CX4945 dosage in vivo, to maybe reach effective picomolar concentrations that would come close to the concentrations used in monoclonal antibody therapy. This could reduce unwanted side effects of an anti-cancer therapy by inhibitors of CK2 [64].

## Supplementary Materials

The following are available online at https://www.mdpi.com/2073-4409/9/5/1138/s1. Figure S1: CK2 does not affect membrane expression of Na^+^/K^+^-ATPase. Supplementary Figure S2: CX4945, but not niclosamide, induces cell death. Supplementary Figure S3: The TMEM16A inhibitor Ani9 inhibits proliferation. Supplementary Figure S4: Inhibition of cell proliferation by different concentrations of CX4945.

## Abbreviations

ATP	adenosine triphosphate
BSA	bovine serum albumin
CaCC	calcium (Ca^2+^)-activated Cl^–^ channel
CFBE	cystic fibrosis bronchial epithelial (cells)
CK2	casein kinase 2
CX4549	silmitasertib
DMSO	dimethyl sulfoxide
ER	endoplasmic reticulum
GPCR	G-protein coupled receptor
HA	hemagglutinin
MTT	3-(4,5-dimethylthiazol-2-yl)-2,5- diphenyl-2H-tetrazolium bromide
PBS	phosphate buffered saline
PM	plasma membrane
RT	room temperature
Scrbld	“scrambled” non-targeting siRNA
TBB	4,5,6,7- tetrabromobenzotriazole
TMEM16A	anoctamin 1
